# Supplementary material for: Scholars as government-appointed research evaluators: Do they create congruence between their professional quality standards and political demands?
Source: PLoS One. 2020 Oct 14;15(10):e0239336. doi: 10.1371/journal.pone.0239336 (PMC7556466; doi:10.1371/journal.pone.0239336)
Supplement: S1 Table — (DOCX) [file pone.0239336.s001.docx]

| **Aggregate Dimension** | **Second-Order Themes** | **First-Order Codes** |
| --- | --- | --- |
| **Motivation for participation** | ***Duty to the academic profession*** | “is part of the professional duties of a university teacher” (G2: 261); a work “which links elements of scientific discourse with elements of self-administration” (G2: 34-35); The “critical evaluation of what one presents as a scientist” (G2: 32) is “a basic component of the scientific discussion” (G2: 32-33) and academic self-administration is “a part of the professional task” (G2: 41-42); that evaluations conducted by academic colleagues are “part of a simulation of competition” (V2: 71) and necessary to “make us better” (V2: 68-69); “If no one evaluates my research for a longer period of time, I will get a colleague to evaluate my research voluntarily. This is a necessary part of scientific competition” (V2: 32-34); performs the activity on the basis of a “commitment to discipline” (V2: 227); “I also use peer review services” (V1: 36-37). |
|  | ***Duty to the public*** | Interviewee V2 states that science is “part of society” (V2: 68) and “it is important to make the best out of the resources one (...) gets” (V2: 72-73); Interviewee V1 states “the taxpayer finances the whole science business and then the taxpayer has a right to have a proper job done (...), according to the motto: Trust is good, but control is better” (V1: 58-59); “In some cases civil servants’ research performance can be improved - to put it mildly” (V1: 59-63). |
|  | ***Protecting research integrity*** | to prevent evaluations from being carried out “by pure science managers” (E2: 36-37), “who (...) do not even know what research really is in today’s world” (E2: 44-48); Acting as an evaluator during politically initiated evaluations prevents “influences from outside science gaining too much weight” (E2: 58-59). |
| **Perception of politically initiated research evaluations** | ***Threat to professional self-regulation*** | “I see the danger that research will be oriented according to what is believed that reviewers would like to hear” (V1: 70-72); “Really revolutionary new ideas could of course get lost, because they have perhaps not yet been published or there is no one on the evaluation committee who recognizes their potential” (E2: 252-254); or “It is difficult to apply research funds with unconventional proposals, because other scholars read the proposal and say: “This is impossible”(E1: 165-168). |
|  | ***Deficits of evaluation procedures*** | “Every evaluation is a very superficial observation of what is happening (...) It never does justice to particularities” (V2: 253-254); “there is certainly much left over that even the attentive evaluator can miss” (G2: 197) and that “an evaluator must be aware of the fact that he cannot look into all peculiarities and niches of one research-area” (G2: 198-200); “The worth of research often only becomes apparent after ten or twenty years. No evaluation can really grasp this long-term perspective” (G1: 352-356); “At first, it does not matter whether I have one hundred or one hundred and fifty publications. The important question is: What was the starting point of these one hundred and fifty or one hundred publications? And: How are they discussed in the field? Are they still discussed?” (G1: 357-360). |
| **Assessing research quality in the role of a researcher** | ***Economists*** | V2 elaborates on his work on migration research, which has shown that “ethnic diversity is good for the economy” (V2: 83) and that migration “is not harmful to the host society” (V2: 84); This work, he further elaborates, is based on “rigorously empirical statistical methods” and delivers “evidence-based (...) policy advice” (V2: 91); “theory, proper empirical verification and then (...) economic policy implications” (V1: 146-148); He points out the work is excellent although it appeared in the German Economic Review “which is not an A-Journal” (V1: 150). |
|  | ***Historians*** | “Actually, just an idea or a sentence, (...) it became independent and then there was a discussion about it” (G1: 92-93); established a new, “very broad understanding of social history” (G2: 66) and “strongly linked historical scholarship and historically oriented social sciences” (G2: 68-69); the “broad comparisons of Germany in international comparison (...) is a second characteristic” (G2: 72-73); “It can take years before a groundbreaking discovery reaches the scientific community. Until then, scholars can be virtually invisible and publish little or nothing” (G1: 101). |
|  | ***Electrical engineers*** | it has led to something “about which the whole scientific world, including reviewers, have initially said: This cannot work” (E1: 155-157); “the first to do so on the basis of mathematical models” (E2: 137-138). |
| **Evaluating research quality in the role of a research evaluator** | ***Number and outlet of publications*** | "number of publications in the field” (E2: 162-164); “Is (research) internationally visible? Do the colleagues publish in the highly ranked journals?” (E2: 204-205); “It is always about: Who publishes in highly ranked journals?” (V2: 142); texts “which can be scaled in some way compared to others” (G1: 205-206); Anyone who “does not write any books in history, in my view, has certain shortcomings” (G1: 301-303); “that is (...) how far things have been taken up internationally by other scholars, influenced their work and the like” (E1: 225-228). |
|  | ***Coherence*** | “a coherent program” (V2: 149) as it is a sign of quality if “there is some kind of center formation, so that one can also achieve something as a group” (V2: 150-151); scholars were able to “present themselves in a certain profile” (G1: 163-164); it was relevant whether “the research profiles are well aligned” (E1: 72-73), because it “makes no sense if everyone does exactly the same thing” (E1: 73-74). |
|  | ***Internationality*** | scholars evaluated “have worked with (...) researchers in Cambridge or Harvard or something like that” (G1: 320-323); researchers had “appropriate international contacts” (E2: 205), “care was taken to ensure that the faculties were then also internationally networked” (V1: 232-233). |
|  | ***Third-party funding*** | third-party funding, which plays a role even though it is “more of a function derived from something else” (V2: 152-153). |
